# Synthesis of water-soluble fluorescent polymeric glycoconjugate for the detection of cholera toxin

**DOI:** 10.1080/15685551.2019.1654695

**Published:** 2019-08-26

**Authors:** Lijuan Feng, Mingjun Zhong, Shizhen Zhang, Min Wang, Zhi-Yong Sun, Qi Chen

**Affiliations:** a Department of Bioengineering, Zunyi Medical University (Zhuhai Compus), Zhuhai, China; b State Key Laboratory of Marine Resource Utilization in South China Sea, Hainan University, Haikou, China; c Institute for Interdisciplinary Research, Jianghan University, Wuhan, China

**Keywords:** Cholera toxin, fluorescence quenching, lactose, sensors, water-soluble conjugated polymer

## Abstract

Considering inherence optical properties of adjoint polyfluorenes and special functions of water-soluble conjugated glycopolymers, a triazole chain glycoconjugate via one-pot method were rapidly synthesized to prepare a lactate ligand polyfluorene with a clear fluorescent label by a nickel-catalyzed Yamamoto coupling polymerization. The water solubility and biocompatibility of the glycoconjugated polymer were ameliorated when the lactose group introduced as the side chain of the conjugated polymer. As a fluorescent multivalent system of glycoconjugates containing pyranogalactose groups, the interaction between pyranogalactose group and cholera toxin B subunit was studied by fluorescence spectrophotometric titration. PF-Lac has a broad application prospect in the check of cholera toxin and the study of glycoprotein interaction.

## 1. Introduction

Some important bioprocesses, such as cell recognition and divergence, cancer transfer, bacterial and virus infections, are closely related to carbohydrate-mediated biological interactions [1–3]. Multivalent interaction, so-called glycoside cluster effect plays a significant role in binding affinity, particularly in those specific biological interactions [4,5]. Glycopolymers are suitable to be the mimic system of glyco-biomacromolecules considering the polyvalence, molecular weight control and molecular structure control [6–8]. These properties have been shown to influence the biological interactions between carbohydrate-carrying chains and their combined targets [9–11].

Cholera is an acute purging infection caused by ingestion of eatables or water polluted by vibrio cholerae, characterized by sudden purging, followed by vomiting; decreased blood pressure, weak pulse, etc. Without appropriate treatment, it can lead to severe vomiting, massive loss of electrolytes such as sodium and potassium in the blood, systemic electrolyte disturbances and even death [12]. In some developing countries, cholera still represents a major potential threat to the health of both human and animals. Cholera give rise to cholera toxin (CT), which has an AB5 hexamer structure. Five identical cholera toxin B subunits (CTB) selectively bind to glycolipid ganglioside GM1 [13]. The CTB can attach to intestinal cells by binding with the pentasaccharide head group of GM1, and this mechanism initiates the disease. Some lactosyl-bearing multivalent systems, such as dendrimers [14,15], nanoparticles [16], and the molecule with aggregation-induced emission feature [17] have been used to study specific combine to CTB. CTB is known to link to lactose by distinguish the end glycolipid section, which is also the end sugar in the GM1 oligosaccharide head [18].

Synthetic fluorescent glycoconjugates have been used as multivalent artificial models of carbohydrate systems and suitable well-defined tools for the study of carbohydrate-based biological events [19–26]. For example, studies on the interaction of carbohydrate-lectin [27] and carbohydrate-bacteria [21] by fluorescent conjugated sugar polymers have been reported. Water-soluble conjugated polymers provide an advantageous platform for the development of new electro-optical devices [28,29], chemical sensors and biosensors [30,31], taking the inherent optoelectronic properties and good water solubility into account. Fluorescent conjugated sugar polymers possessing fluorescent backbones and carbohydrate-binding moieties are used in the investigation of carbohydrate-mediated bio-interaction and biosensor applications because of their water solubility, their unique optical properties and their high sensitivity to external stimuli and good biocompatibility [32,33].

It is well known that polyfluorenes exhibit intrinsic optical properties [34] and have been used as various sensors [35]. Herein, a lactosyl-bearing polyfluorene was synthesized and characterized, the latent application of the CT fluorescence sensor is also investigated. The introduction of lactose part can improve the solubility of water and biocompatibility of the polyfluorene system. Moreover, lactose moieties can provide CTB-oriented binding sites via specific biological interactions, thus, enhance the binding affinity via multivalent interactions, which leads to aggregation of lactosyl-bearing polyfluorene and significant fluorescence quenching.

## 2. Experiment

### 2.1 Materials

Peracetylated lactose (1) and HBr (33% solution in acetic acid) were commercially available from Acros Scientific Co. Ltd. Tetrabutylammonium hydrogen sulfate and sodium azide were commercially available from J&K Scientific Co. Ltd. 1,5-cyclooctadiene (1,5-COD), bis(1,5-cyclooctadiene)nickel(0) (1,5-COD-Ni）and 2,2ʹ-bipyridyl (2,2ʹ-Bpy) were bought from Aldrich Chemicals. Sodium methoxide and sodium ascorbate were purchased from Shanghai Chemical Technology Co. Ltd. CTB, bovine serum albumin (BSA) and phosphate buffer solution (PBS, 50 mM, pH 7.3) were bought from Sigma-Aldrich Co. Ltd. N, N-Dimethylformamide (DMF), dichloroethane (DCE), chloroform, methanol and tetrahydrofuran (THF) were dried before use. Unless otherwise stated, Other chemical reagents can be bought commercially and used as they are. The Millipore Purification System (Mily Q Water) was used to purify deionized water. 9,9-bis(4-propargyloxy phenyl)-2,7-dibromo fluorene (**2**) was prepared according to reported methods [36].

### 2.2 Instrumental characterization and structural analysis

Measurement of Nuclear Magnetic Resonance (NMR) spectra (^1^H and ^13^C) were on Bruker DMX400 NMR spectrometer (Bruker Daltonics Inc., Germany). VG PLATFORM mass spectrometer was applied to record mass spectra by electrospray ionization (ESI (+)) (VG Elemental, England). The infrared (IR) spectra of small molecules and polymers were recorded with KBr pellets on a Perkin–Elmer Paragon 1000 Fourier transform infrared (FTIR) spectrometer (PerkinElmer Instruments Co. Ltd., USA). Perkin Elmer Lamda 900 UV-vis-NIR spectrophotometer (Perkin Elmer Instruments Co. Ltd.) was used to record UV-vis spectra by a quartz pool with a path length of 1 cm. Measurement of Fluorescence Spectra were used by Perkin Elmer LS55 Luminescence Spectrometer (PerkinElmer Instruments Co. Ltd., USA) at 1 cm Path Length. Screening molecular mass of polymers dissolved in THF was used by Agilent 1100 GPC-SEC Analysis System (Agilent Technologies, USA). The average molecular weight and molecular weight (Mn and Mw) of polystyrene were reckoned by calibration curve of polystyrene reference material.

### 2.3 Synthesis of monomer 3 via one-pot reactions

For the solution of acetylated lactose 1 (1.0 g, 1.5 mmol) in dry chloroform (15 mL), HBr (33% solution in acetic acid, 2.0 mL) was added dropwise in an ice water bath. Stirring the liquor at room temperature was for about 6 h under nitrogen protection until the reaction was accomplished. The volatile compounds are removed by decompression, and chloroform (10 mL), saturated baking soda solution (2.0 M, 5 mL), sodium azide (0.16 g, 4.6 mmol) and tetrabutylammonium bisulfate (0.5 g, 3.0 mmol) were added sequentially. Two hours later, 9,9-bis(4-propargyloxy phenyl)-2,7-dibromo fluorene 2 (292 mg, 1.0 mmol), CuSO_4_ · 5H_2_O (12.5 mg, 0.1 mmol), L-ascorbic acid Sodium Salt (30 mg, 0.30 mmol) and alcohol (5 mL) were added to the solution. The reaction mixture was kept stirring at room temperature for about 16 h. After removing solvent in vacuum, water (20 mL) was added to extract the product with ethyl acetate (170 mL). The merged organic layer was dehydrated with anhydrous Na_2_SO_4_ and evaporated in vacuum. Column chromatography (ethyl acetate-light petroleum, 2:1) was used for purification of the product 3 as foamed solid (0.9 g, 31%). ^13^C NMR (100 MHz, CDCl_3_): *δ* 19.2, 19.4, 19.5, 19.6, 19.8, 19.9, 20.1, 59.5, 59.5, 60.8, 61.0, 63.5, 65.6, 68.2, 69.6, 70.0, 71.7, 74.6, 75.0, 84.6, 100.2, 113.9, 120.3, 121.0, 128.3, 123.0, 136.2, 136.8, 143.9, 152.5, 156.3, 168.2, 168.5, 169.1, 169.2, 169.3, 169.5. ^1^H NMR (400 MHz, CDCl_3_): *δ* 1.77 (s, 6H), 1.95 (s, 6H), 2.01 (s, 6H), 2.03 (s, 6H), 2.05 (s, 6H), 2.06 (s, 6H), 2.14 (s, 6H), 3.95–3.87 (m, 6H), 4.15–4.06 (m, 6H), 4.46 (d, *J* = 10.9 Hz, 2H), 4.93 (dd, *J* = 2.9, 9.9 Hz, 2H), 5.08 (d, *J* = 8.2 Hz, 2H), 5.13 (s, 4H), 5.40–5.34 (m, 6H), 5.83 (d, *J* = 9.8 Hz, 2H), 6.83 (d, *J* = 9.0 Hz, 4H), 7.04 (d, *J* = 8.6 Hz, 4H), 7.45–7.43 (m, 4H), 7.55 (d, *J* = 9.1 Hz, 2H), 7.76 (s, 2H). ESI(+)-MS: calcd. for C_83_H_90_O_36_N_6_Br_2_: 1907.45 [M]; found 1946.86 [M + K]^+^.

### 2.4 Synthesis of polymer PF-AcLac by the Yamamoto coupling polymerization

Dry 1,5-cyclooctadiene (0.052 mL, 0.42 mmol) was put in anhydrous DMF (15 mL) solutions of 1,5-COD-Ni (112 mg, 0.41 mmol) and 2,2ʹ-Bpy (64 mg, 0.41 mmol). The mixture was heated and stirred at 80°C for 1 h, and sugar-bearing monomer 3 (286 mg, 0.15 mmol) was added to the purple solution. The mixture was heated and stirred at 80°C under the nitrogen atmosphere for 24 h. The resulting polymer was precipitated by pouring the reaction mixture into a methanol-water solution. PF-AcLac was isolated as grey powder (200 mg, 78%). ^1^H NMR (400 MHz, CDCl_3_): *δ* 1.77 (bs, 6H), 1.99 (bs, 6H), 2.05 (bs, 24H), 2.14 (bs, 6H), 3.92 (br, 6H), 4.10 (br, 6H), 4.44–4.53 (m, 4H),4.95–4.98 (m, 2H), 5.01–5.14 (m, 6H), 5.34–5.39 (m, 6H), 5.81 (br, 2H), 6.82–6.88 (m, 4H), 7.04–7.20 (m, 3H),7.45–7.51 (m, 3H),7.56–7.63 (m, 4H), 7.75 (bs, 2H). ^13^C NMR (100 MHz, CDCl_3_): *δ* 19.0, 19.5, 19.7, 19.8, 59.8, 60.6, 60.8, 65.6, 68.0, 69.5, 69.7, 69.9, 71.6, 74.7, 74.8, 76.1, 84.6, 100.1, 113.4, 113.8, 120.1, 126.0, 126.4, 128.4, 130.7, 136.8, 137.7, 143.9, 152.5, 156.4, 168.1, 168.3, 168.5, 169.0, 169.1, 169.2, 169.3. IR: ν (cm^–1^): 2938 (w), 1752 (s), 1593 (m), 1513 (m), 1482 (s), 1372 (m), 1321 (m), 1235 (s), 1111 (s), 809 (m). GPC (THF, polystyrene standard): *M*_n_ = 28, 600 g/mol; polydispersity = 1.74.

### 2.5 Synthesis of polymer PF-Lac

CH_3_ONa (1.0 M methanol solution) was added to the protected sugar polymer PF-AcLac (150 mg) in CH_2_Cl_2_/CH_3_OH (v/v = 1:3, 20 mL) solution drop by drop until the solution pH reached 11. The reaction mixture was stirred at room temperature overnight. The solid was gathered by centrifugation at 8000 rpm and washed twice with CH_2_Cl_2_. After the solvent is removed under diminished pressure, the residue was diluted with 10 ml water. The obtained solution was placed in a cellulose dialysis tube (with a trapped molecular weight of 3,500), dialyzed with water for 3 days, and freeze-dried to obtain the required solid polymer PF-Lac (95.5 mg, 94%). IR: ν (cm^–1^): 3421 (bs), 2934 (w), 1590 (m), 1513 (m), 1479 (s), 1325 (m), 1103 (s), 804 (m). ^1^H NMR (400 MHz, DMSO-*d*_6_): *δ* 3.31–3.44 (m, 6H), 3.51–3.73 (m, 14H), 3.75–3.88 (m, 4H), 4.28 (br, 2H), 5.11 (s, 4H), 5.68 (br, 2H), 6.95–7.04 (m, 4H), 7.15–7.24 (m, 2H), 7.25–7.70 (m, 4H), 7.75–8.10 (m, 4H), 8.44 (bs, 2H), ^13^C NMR (100 MHz, DMSO-*d*_6_): *δ* 60.0, 60.9, 68.2, 70.6, 71.9, 73.3, 75.2, 75.6, 77.7, 79.2, 79.7, 86.9, 103.5, 114.7, 123.7, 125.1, 126.5, 127.3, 128.7, 131.7, 134.3, 136.4, 139.2, 142.5, 157.2. GPC (THF, polystyrene standard): *M*_n_ = 19, 900 g/mol; polydispersity = 1.71.

### 2.6 Studies of the interactions between PF-Lac and CTB by fluorometric titration analysis

PF-Lac stock solution was prepared in PBS aqueous solution (50 mM, pH 7.3). Equivalent CTB (2 μL) was added to the same PBS buffer and PF-Lac solution (1.5 × 10^–6^ M, 2.0 mL). The final concentration of CTB was ranged from 1.0 × 10^−8^ to 8.0 × 10^−8^ M. After each addition, the samples were balanced overnight and the spectra were recorded. The excitation wavelength was 320 nm and the emission scan ranged from 310 to 630 nm.

### 2.7 Studies on the selectivity of fluorometric detection for CTB

Preparation of PF-Lac stock solution was in PBS (50 mM, pH 7.3). BSA (2 μL) and CTB (2 μL) fractions in alike PBS buffer were injected in PF-Lac solution (1.5 × 10^–6^ M, 2.0 mL) subsequently. After each addition, the sample was balanced overnight and the spectra were recorded. The final concentrations of BSA and CTB were 8.0 × 10^−8^ M, respectively. The excitation wavelength is 317 nm and the radiate scanning range is 330–630 nm.

## 3. Results and discussion

### 3.1 Synthesis and characterization of monomer and polymers

**Scheme 1.**
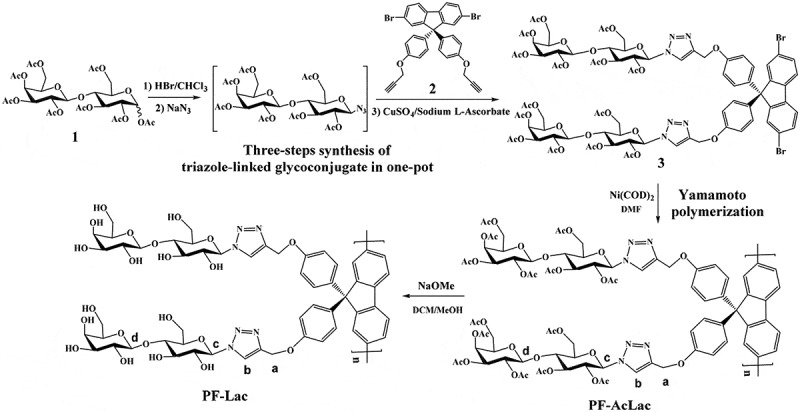
Synthetic routes to the monomer 3 and polymer PF-Lac.

The synthetic routes of monomers and sugar polymers are outlined in Scheme 1. For monomer preparation, a rapid one-pot synthetic method to triazole-linked glycoconjugate from commercially available sugar acetate was used [37,38], which includes bromination, azidation and Cu(I)-catalyzed Huisgen 1,3-dipole cyclic addition reaction. As shown in Scheme 1, peracetylated lactose was used as the starting material for this method. After bromination with HBr in acetic acid and removal of all volatiles of solvents and acids, subsequent azide conversion can furnish intermediate acetylated lactosyl azide. 1,3-Dipolar selective ligation between propargyl fluorene derivative 2 and sugar azide intermediate catalyzed by in situ copper (I) are easy to produce lactose-containing monomer 3. A signal peak at 7.76 ppm (2H) in the ^1^H NMR spectrum as well as two peaks at 120.6 ppm and 144.0 ppm in the ^13^C NMR spectrum confirmed the formation of the triazole ring.

**Figure 1.**
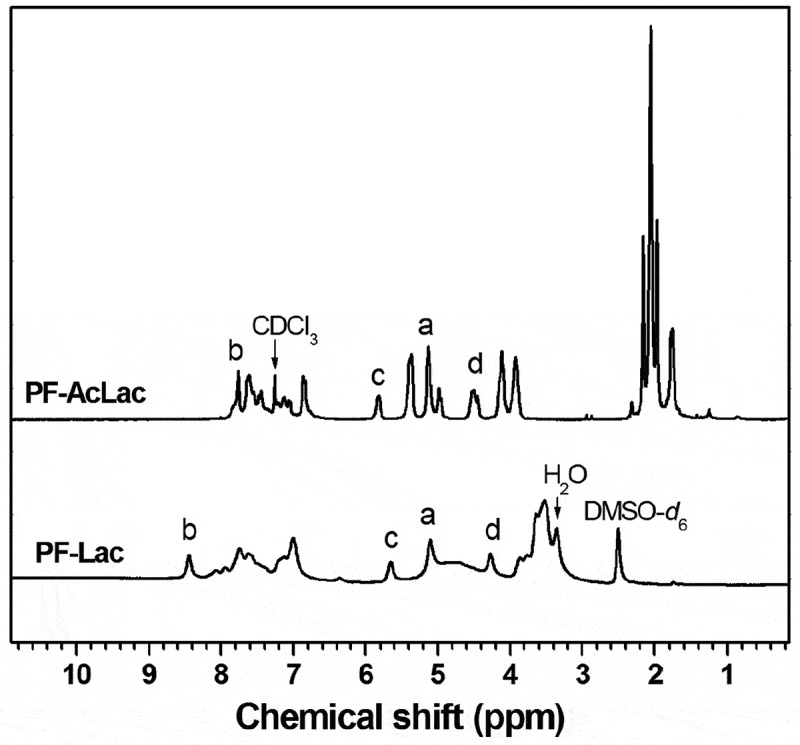
^1^H NMR spectra of polymer PF-AcLac (in CDCl_3_) and polymer PF-Lac (in DMSO-*d*_6_).

The Ni-catalyzed Yamamoto coupling polymerization of monomer 3 can afford a well-defined and protected glycopolymer, PF-AcLac, which is a poly(fluorene) containing the side-chain lactosyl derivatives. The PF-AcLac was treated with CH_3_ONa/CH_3_OH to deprotect all acetyl groups. The desired water-soluble conjugated glycopolymers (PF-Lac) were obtained in excellent yield (94%). The ^1^H and ^13^C NMR spectra of polymers PF-AcLac and PF-Lac are shown in Figure 1 and the supporting information, which can prove the successful preparation of the desired polymers. There are two reasons for HNMR signal of proton b down-field shifted significantly from PF-AcLac to PF-Lac. One is that the chemical environment of proton b is changed due to fully de-acetylation from PF-AcLac to PF-Lac. The more important is that the solvent for HNMR study is different from PF-AcLac to PF-Lac. For PF-AcLac, solvent is CDCl_3_. While for PF-Lac, solvent is DMSO-*d*_6_. The potential hydrogen bond acceptor would lead to shift the proton signal downfield [39]. Selection of 9,9-bis (4-hydroxyphenyl) fluorene as polymeric monomers is beneficial to provide more effective shielding effect on the polyfluorenyl skeleton, because there is a non-flexible phenylene spacer between the main chain of the polymer and the side chain of the sugar, which can inhibit the formation of aggregates or receptors [40].

**Figure 2.**
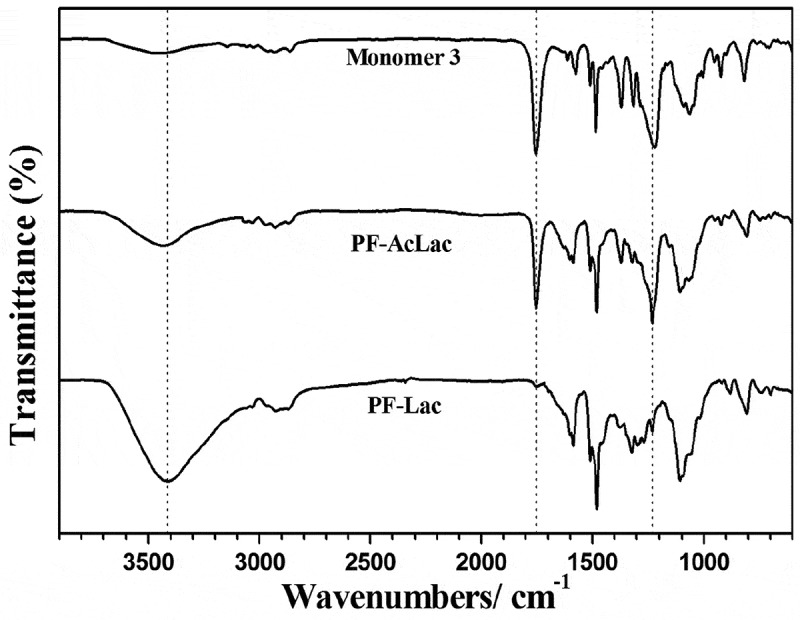
IR spectra of monomer 3, polymer PF-AcLac, and polymer PF-Lac.

FT-IR spectra demonstrated the successful polymerization and deacetylation of conjugated glycopolymers. As shown in Figure 2, absorption bands at 2850–2950 cm^–1^ in the FT-IR spectra of the monomer, polymers PF-AcLac and PF-Lac represent CH stretching from alkyl chains of monomers. After treating PF-AcLac with CH_3_ONa/CH_3_OH, the bands at 1750 cm^–1^ (C = O) and 1200 cm^–1^ (C–O stretching in ester) of PF-AcLac disappeared and confirmed perdeacetylation. Moreover, in the FT-IR spectra, there is a wide absorption band at 3400 cm^−1^ of polymer PF-Lac showed the extension of –OH, which are the free hydroxyl groups on the sugar part. This information also proves clearly that the polymerization of sugar-containing aromatic monomer is a good way to prepare well-defined conjugated glycopolymers.

**Figure 3.**
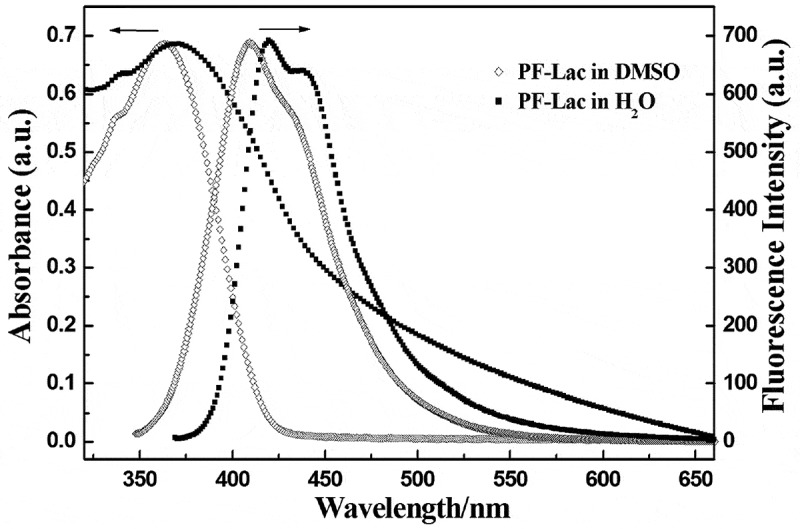
UV–vis absorption and fluorescence spectra of polymer PF-Lac in DMSO and water.

PF-AcLac is soluble in general solvents, such as CHCl_3_, CH_2_Cl_2_, acetyl acetate and THF, but not soluble in methanol and water. Gel permeation chromatography (GPC) analysis of polystyrene standard materials indicate that the number average molecular weight (Mn) of PF-AcLac is 28,600 g/mol with polydispersity of 1.74. After deprotection, the solubility of glycopolymer PF-Lac was different from its precursor. PF-Lac is soluble in water medium and even better in DMF and DMSO. Moreover, it exhibits intrinsic optical properties. For the PF-Lac in DMSO solution, the maximum absorption and emission peaks were found at 366 nm and 415 nm, and the vibration peak at 433 nm. These peaks are due to the π-π* transition of the main chain of polyfluorene. In aqueous solution, the maximum absorption peak of PF-Lac is at 378 nm, the maximum emission peak is at 424 nm and the vibration peak is at 442 nm. As shown in Figure 3, we can see that the absorption and emission spectra of conjugated glycopolymer PF-Lac in water exhibit a significant red shift compared with the polymer in DMSO solution, because the planar conformation or aggregation of the polymer backbone is enhanced due to increased solvent polarity [41].

### 3.2 Fluorescence ‘turn-off’ detection of CT

**Scheme 2.**
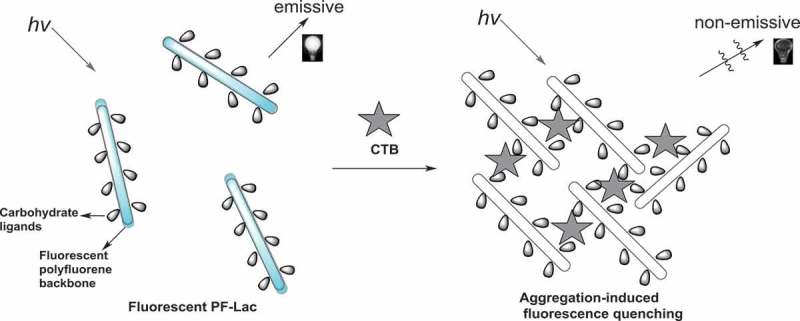
Schematic representation of fluorescence ‘turn-off’ assay for CTB by PF-Lac.

As mentioned above, CT consists of the five same monomers with a galactose binding site of GM1 ganglioside. Therefore, various types of molecules containing identical terminal galactose groups can be designed to bind to CTB. Lactose composition of amylaceum and a terminus-galatosum unit are usually used to design CT transducer. Water-soluble PF-Lac, possessing both a polyfluorene backbone and many lactosyl units, shows high photoluminescence in water. We can assume that when CTB was added into the dilute water solution of PF-Lac, the binding of CTB with polylactose-attached polyfluorene occurred, leading to the aggregation of PF-Lac. Therefore, the fluorescence of PF-Lac is quenched significantly due to aggregation, which can be used for CTB sensing. Schematic representation of fluorescence ‘turn-off’ assay for CTB by PF-Lac was shown in Scheme 2.

**Figure 4.**
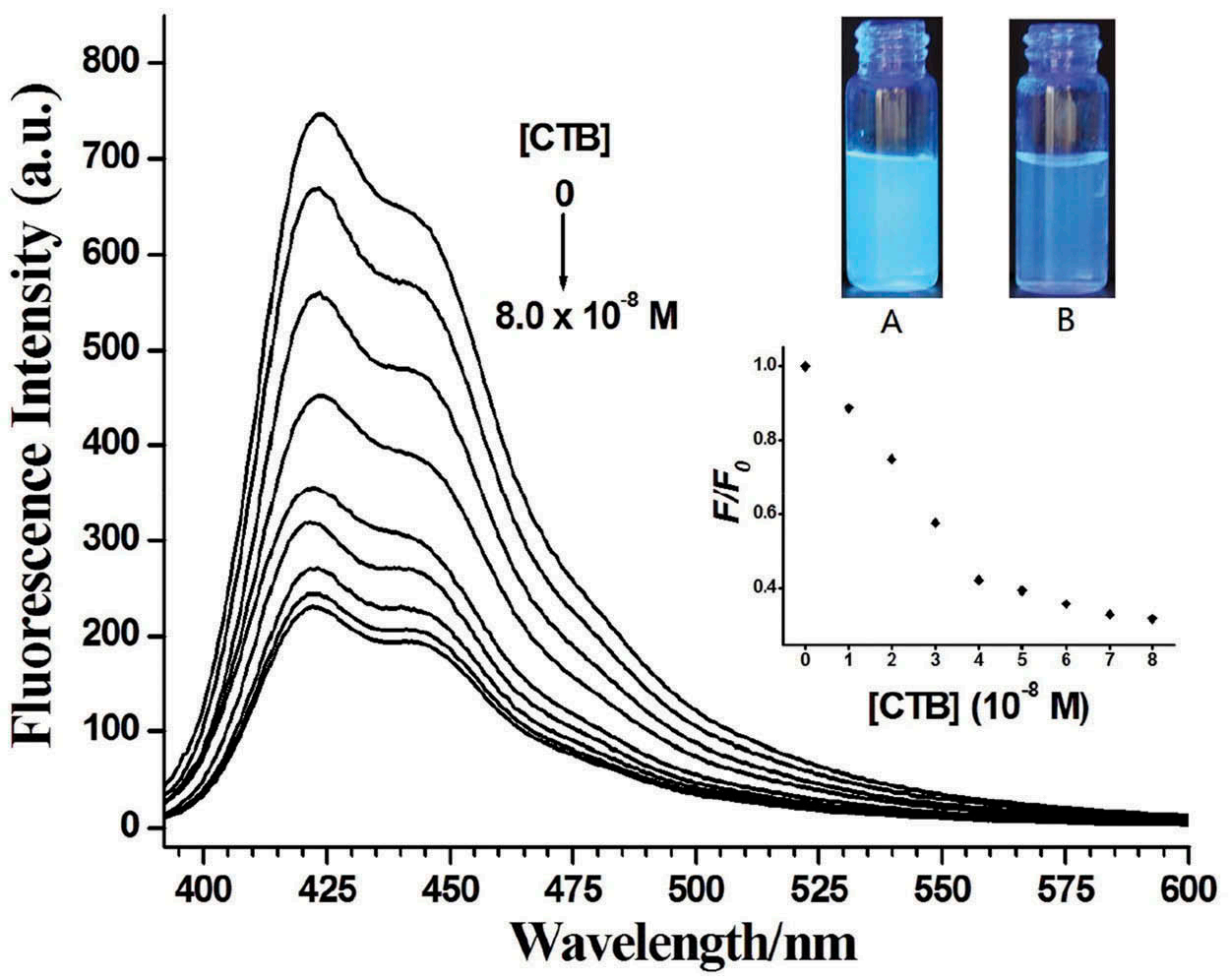
Fluorescence spectra of polymer PF-Lac (1.5 × 10^–6^ M) in the absence and presence of various concentrations of CTB in PBS solution at room temperature. CTB concentrations from top to bottom are 0, 1.0, 2.0, 3.0, 4.0, 5.0, 6.0, 7.0 and 8.0 × 10^−8^ M (Inset displays the photos of the corresponding solutions of PF-Lac in the absence (A) and presence (B) of CTB (8.0 × 10^−8^ M) under UV light (365 nm) illumination).

The interaction between PF-Lac and CTB was studied by detecting the fluorescence intensity of PF-Lac in the presence of CTB in PBS. As shown in Figure 4, when CTB (0–8.0 × 10^–8^ M) was gradually added to the solution of PF-Lac (1.5 × 10^–6^ M) in the PBS (50 mM, pH = 7.3), most of fluorescence was quenched. The possible mechanism of fluorescence quenching of PF-Lac is attributed to the aggregation of PF-Lac/CTB binding, resulting in self-quenching. In static quenching, the quencher forms a ground-state complex with the fluorophore, and a linear Stern–Volmer relationship (F_0_/F = 1+ K_SV_[Q]) occurs [25,42]. K_SV_, as the Stern–Volmer constant, indicates the efficiency of quenching. The Stern–Volmer quenching constant of polymer PF-Lac by CTB was calculated as 1.6 × 10^7^ M^−1^ based on the linear part of the curve (Figure S1).

**Figure 5.**
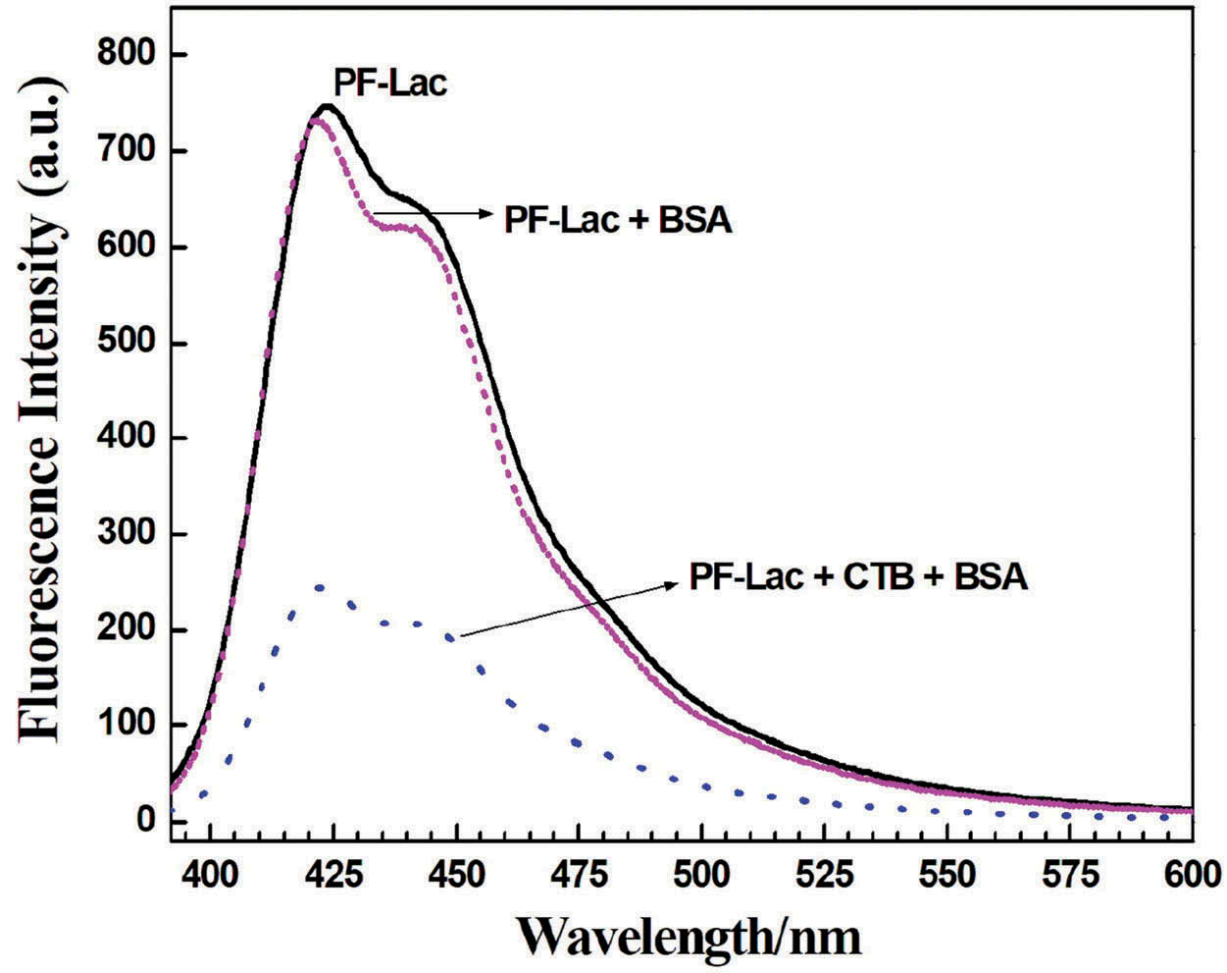
Fluorescence spectra of polymer PF-Lac, polymer PF-Lac with the addition of BSA, and polymer PF-Lac following the addition of BSA and CTB at room temperature.

To study the selectivity of fluorescence check for CTB and the specificity of carbohydrate–protein interaction, the PF-Lac assay was conducted with other bio-macromolecules. We can see that the photoluminescence intensity of PF-Lac did not change significantly at presence of the BSA protein under the same conditions (Figure 5). In addition, when PF-Lac was presented with the same amount of CTB and BSA in PBS solution, the intensity of fluorescence quenching was almost identical as that observed in the solution of CTB. These results revealed that PF-Lac shows great potential use in the selective detection of biomacromolecules.

## 4. Conclusion

Fluorescent conjugated polyfluorene with side-chain lactopyranosyl ligand (PF-Lac) was synthesized by one-pot formation of triazole-linked glycoconjugates and Yamamoto C-C coupling polymerization. Using fluorescent fluorene scaffolds and sugar-binding ligands, this well-defined fluorescent glycoconjugate polymer is used to study the interaction of CTB subunits by fluorescence spectrophotometric titration. When CTB is presented in deliquate PF-Lac aqueous solution, the binding of CTB with polyfluorene containing multi-lactose groups occurred, which cause the accumulation of PF-Lac. Therefore, the fluorescence of PF-Lac is quenched due to self-aggregation. We believe that these methods for insight into carbohydrate-mediated biological interactions have shown potential applications in the sensing of biomacromolecules and the glycobiology study.

## References

[1] Krasnova L, Wong CH. Understanding the chemistry and biology of glycosylation with glycan synthesis. Annu Rev Biochem. 2016;85:599–630. 27145845 10.1146/annurev-biochem-060614-034420

[2] Sun X, James TD Glucose sensing in supramolecular chemistry. Chem Rev. 2015;115:8001–8037. 25974371 10.1021/cr500562m

[3] Pinho SS, Reis CA Glycosylation in cancer: mechanisms and clinical implications. Nat Rev Cancer. 2015;15:540–555. 26289314 10.1038/nrc3982

[4] Mueller C, Despras G, Lindhorst TK Organizing multivalency in carbohydrate recognition. Chem Soc Rev. 2016;45:3275–3302. 27146554 10.1039/c6cs00165c

[5] Mooibroek TJ, Casas-Solvas JM, Harniman RL, et al A threading receptor for polysaccharides. Nat Chem. 2016;8:69–74. 26673266 10.1038/nchem.2395 PMC5856347

[6] Appelhans D, Klajnert-Maculewicz B, Janaszewska A, et al Dendritic glycopolymers based on dendritic polyamine scaffolds: view on their synthetic approaches, characteristics and potential for biomedical applications. Chem Soc Rev. 2015;44:3968–3996. 25519948 10.1039/c4cs00339j

[7] Chen Y, Lord MS, Piloni A, et al Correlation between molecular weight and branch structure of glycopolymers stars and their binding to lectins. Macromolecules. 2015;48:346–357.

[8] Park H, Rosencrantz RR, Elling L, et al Glycopolymer brushes for specific lectin binding by controlled multivalent presentation of *N-*acetyllactosamine glycan oligomers. Macromol Rapid Comm. 2015;36:45–54. 10.1002/marc.201400453 25354386

[9] Lin K, Kasko AM Carbohydrate-based polymers for immune modulation. ACS Macro Lett. 2014;3:652–657. 25844272 10.1021/mz5002417 PMC4372078

[10] Yilmaz G, Becer CR Glyconanoparticles and their interactions with lectins. Polym Chem. 2015;6:5503–5514.

[11] Wibowo A, Peters EC, Hsieh-Wilson LC Photoactivatable glycopolymers for the proteome-wide identification of fucose-α(1-2)-galactose binding proteins. J Am Chem Soc. 2014;136:9528–9531. 24937314 10.1021/ja502482a PMC4105059

[12] Burki T End of a cholera epidemic in South Sudan declared. Lancet. 2018;391:647. 29617255 10.1016/S0140-6736(18)30261-7

[13] Mertz JA, McCann JA, Picking WD Fluorescence analysis of galactose, lactose, and fucose interaction with the cholera toxin B subunit. Biochem Biophys Res Commun. 1996;226:140–144. 8806604 10.1006/bbrc.1996.1323

[14] Delbianco M, Bharate P, Varela-Aramburu S, et al Carbohydrates in supramolecular chemistry. Chem Rev. 2016;116:1693–1752. 26702928 10.1021/acs.chemrev.5b00516

[15] Dohi H, Kanazawa T, Saito A, et al Bis(β-lactosyl)-[60]fullerene as novel class of glycolipids useful for the detection and the decontamination of biological toxins of the Ricinus communis family. Beilstein J Org Chem. 2014;10:1504–1512. 25161707 10.3762/bjoc.10.155 PMC4142837

[16] Calavia PG, Chambrier I, Cook MJ, et al Targeted photodynamic therapy of breast cancer cells using lactose-phthalocyanine functionalized gold nanoparticles. J Colloid Interf Sci. 2018;512:249–259. 10.1016/j.jcis.2017.10.030 29073466

[17] Hu X-M, Chen Q, Wang J-X, et al Tetraphenylethylene-based glycoconjugate as a novel fluorescence “turn-on” sensor for cholera toxin. Chem-Asian J. 2011;6:2376–2381. 21748854 10.1002/asia.201100141

[18] Turnbull WB, Precious BL, Homans SW Dissecting the cholera toxin-ganglioside GM1 interaction by isothermal titration calorimetry. J Am Chem Soc. 2004;126:1047–1054. 14746472 10.1021/ja0378207

[19] Zeng X, Qu K, Rehman A Glycosylated conductive polymer: a multimodal biointerface for studying carbohydrate–protein interactions. Acc Chem Res. 2016;49:1624–1633. 27524389 10.1021/acs.accounts.6b00181

[20] Martos-Maldonado MC, Casas-Solvas JM, Quesada-Soriano I, et al Poly(amido amine)-based mannose-glycodendrimers as multielectron redox probes for improving lectin sensing. Langmuir. 2013;29:1318–1326. 23286545 10.1021/la304107a

[21] Seto H, Kamba S, Kondo T, et al Metal mesh device sensor immobilized with a trimethoxysilane-containing glycopolymer for label-free detection of proteins and bacteria. ACS Appl Mater Interfaces. 2014;6:13234–13241. 25014128 10.1021/am503003v

[22] Ma F, Rehman A, Liu H, et al Glycosylation of quinone-fused polythiophene for reagentless and label-free detection of *E. coli*. Anal Chem. 2015;87:1560–1568. 25569130 10.1021/ac502712q

[23] Chen Q, Bian N, Cao C, et al Glucosamine hydrochloride functionalized tetraphenylethylene: a novel fluorescent probe for alkaline phosphatase based on the aggregation-induced emission. Chem Commun. 2010;46:4067–4069. 10.1039/c002894k 20454747

[24] Chen Q, Han B-H Prepolymerization and postpolymerization functionalization approaches to fluorescent conjugated carbazole-based glycopolymers via “click chemistry”. J Polym Sci Part A: Polym Chem. 2009;47:2947–2958.

[25] Chen Q, Xu Y, Du Y, et al Triphenylamine-based fluorescent conjugated glycopolymers: synthesis, characterization and interactions with lectins. Polymer. 2009;50:2830–2835.

[26] Shie J, Liu Y, Hsiao J, et al A cell-permeable and triazole-forming fluorescent probe for glycoconjugate imaging in live cells. Chem Commun. 2017;53:1490–1493. 10.1039/c6cc08805h 28084480

[27] Sun P, He Y, Lin M, et al Glyco-regioisomerism effect on lectin-binding and cell-uptake pathway of glycopolymer-containing nanoparticles. ACS Macro Lett. 2014;3:96–101. 10.1021/mz400577p 35651117

[28] Okamoto K, Zhang J, Housekeeper JB, et al C–H arylation reaction: atom efficient and greener syntheses of π-conjugated small molecules and macromolecules for organic electronic materials. Macromolecules. 2013;46:8059–8078.

[29] Chen X, Liu H, Xu Z, et al Highly regiosymmetric homopolymer based on dioxythiophene for realizing water-processable blue-to-transmissive electrochrome. ACS Appl Mater Interfaces. 2015;7:11387–11392. 25943774 10.1021/acsami.5b01908

[30] Gorityala BK, Lu Z, Leow ML, et al Design of a “turn-off/turn-on” biosensor: understanding carbohydrate-lectin interactions for use in noncovalent drug delivery. J Am Chem Soc. 2012;134:15229–15232. 22935034 10.1021/ja306288p

[31] Cecioni S, Imberty A, Vidal S Glycomimetics versus multivalent glycoconjugates for the design of high affinity lectin ligands. Chem Rev. 2015;115:525–561. 25495138 10.1021/cr500303t

[32] Wang K, Zhang XY, Zhang XQ, et al Fluorescent glycopolymer nanoparticles based on aggregation-induced emission dyes: preparation and bioimaging applications. Macromol Chem Phys. 2015;216:678–684.

[33] Chen Q, Cui Y, Zhang T-L, et al Fluorescent conjugated polyﬂuorene with pendant lactopyranosyl ligands for studies of Ca^2+^-mediated carbohydrate- carbohydrate interaction. Biomacromolecules. 2010;11:13–19. 20000350 10.1021/bm901165n

[34] Liang J, Cui J, Guo T, et al Highly efficient blue polyfluorenes using blending materials as hole transport layer. Org Electron. 2017;51:111–118.

[35] Cingil HE, Storm IM, Yorulmaz Y, et al Monitoring protein capsid assembly with a conjugated polymer strain sensor. J Am Chem Soc. 2015;137:9800–9803. 26230158 10.1021/jacs.5b05914

[36] Chen Q, Cheng Q-Y, Zhao Y-C, et al Glucosamine hydrochloride functionalized water-soluble conjugated polyfluorene: synthesis, characterization, and interactions with DNA. Macromol Rapid Commun. 2009;30:1651–1655. 21638433 10.1002/marc.200900226

[37] Chittaboina S, Xie F, Wang Q One-pot synthesis of triazole-linked glycoconjugates. Tetrahedron Lett. 2005;46:2331–2336.

[38] Lim D, Brimble MA, Kowalczyk R, et al Protecting-group-free one-pot synthesis of glycoconjugates directly from reducing sugars. Angew Chem Int Edit. 2014;53:11907–11911. 10.1002/anie.201406694 25199905

[39] Gottlieb HE, Kotlyar V, Nudelman A NMR chemical shifts of common laboratory solvents as trace impurities. J Org Chem. 1997;62:7512–7515. 11671879 10.1021/jo971176v

[40] Marsitzky D, Vestberg R, Blainey P, et al Self-encapsulation of poly-2,7-fluorenes in a dendrimer matrix. J Am Chem Soc. 2001;123:6965–6972. 11459474 10.1021/ja010020g

[41] Xue C, Donuru VR, Liu H Versatile prepolymerization and postpolymerization functionalization approaches for well-defined fluorescent conjugated fluorene-based glycopolymers. Macromolecules. 2006;39:5747–5752.

[42] Lakowicz JR Principles of fluorescence spectroscopy. 3rd ed. New York: Springer; 2006.

