# Supplementary material for: Synthesis of water-soluble fluorescent polymeric glycoconjugate for the detection of cholera toxin
Source: Des Monomers Polym. 2019 Aug 26;22(1):150–8. doi: 10.1080/15685551.2019.1654695 (PMC6719259; doi:10.1080/15685551.2019.1654695)
Supplement: Supplemental Material [file TDMP_A_1654695_SM6988.pdf]

**Supporting Information** for

# Synthesis of Water-Soluble Fluorescent Polymeric Glycoconjugate for the Detection of Cholera Toxin

Lijuan Feng[a]*, Mingjun Zhong[b], Shizhen Zhang[c], Min Wang[a], Zhi-Yong Sun[a], Qi Chen[b]*

[a] *Department of Bioengineering, Zunyi Medical University (Zhuhai Compus), Zhuhai 519041, China*

[b] *State Key Laboratory of Marine Resource Utilization in South China Sea, Hainan University, Haikou 570228, China*

[c] *Institute for Interdisciplinary Research, Jianghan University, Wuhan 430056, China*

*Corresponding authors. E-mail: fljcyczh@126.com(L.Feng), chenqi@hainanu.edu.cn (Q. Chen).

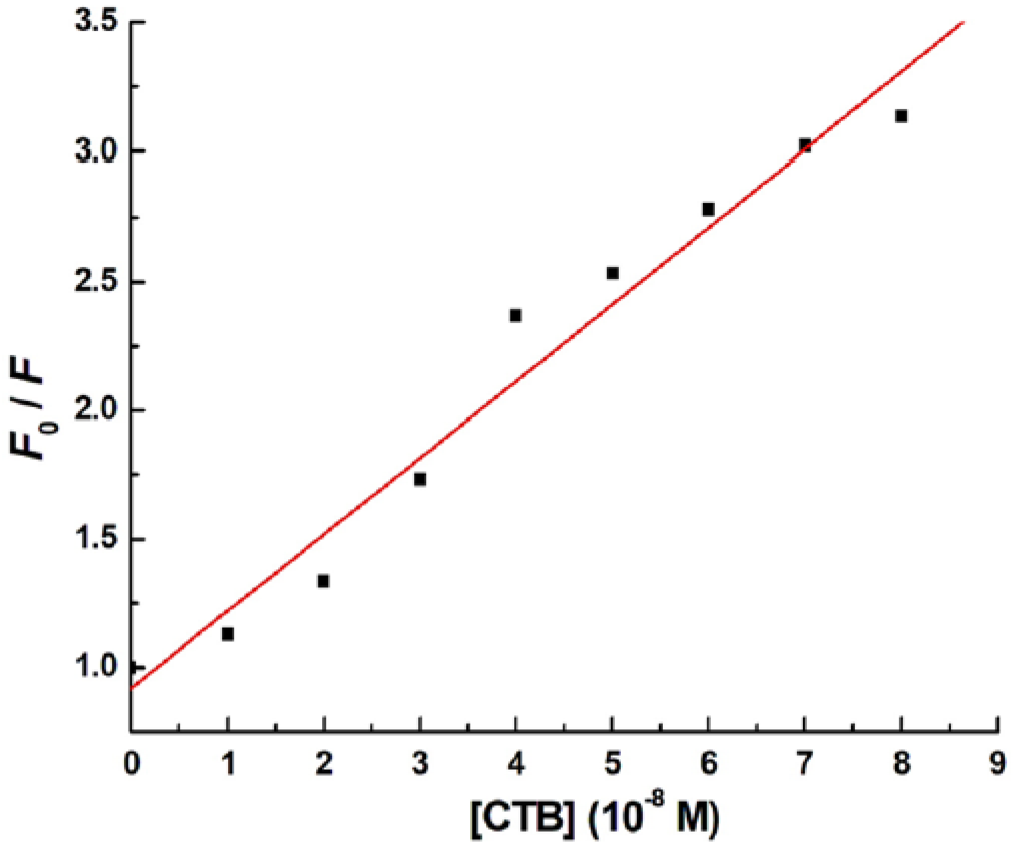


Figure S1. The changed fluorescent intensity as a function of quencher CTB concentration ($F_0$ is the fluorescent intensity in the absence of quencher, F is the fluorescent intensity as a function of quencher concentration [CTB]).

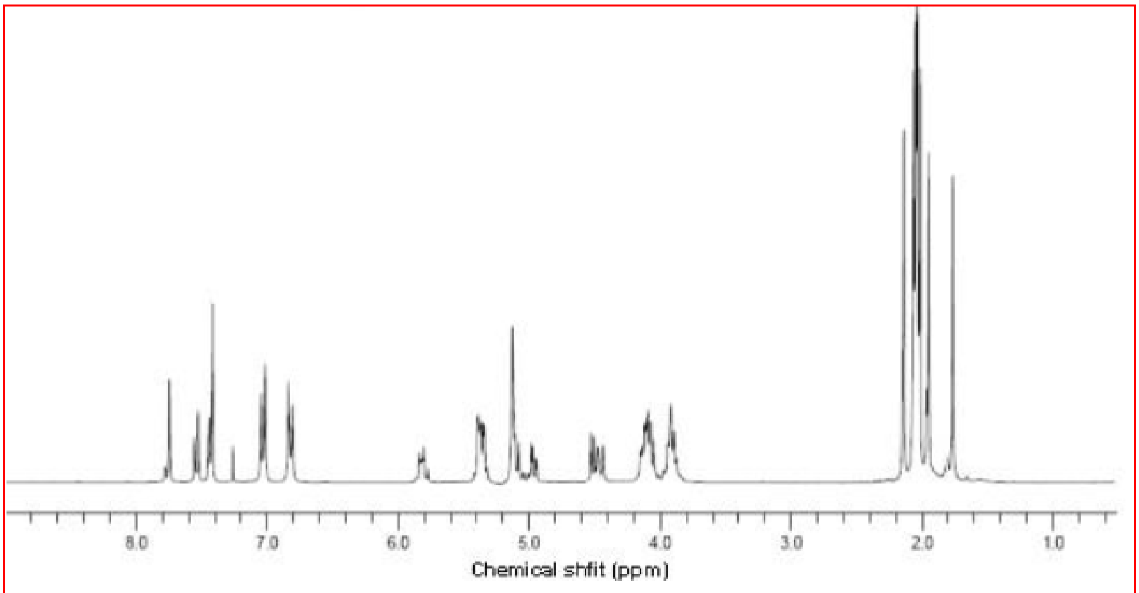


$^1$H NMR spectrum of monomer **3**

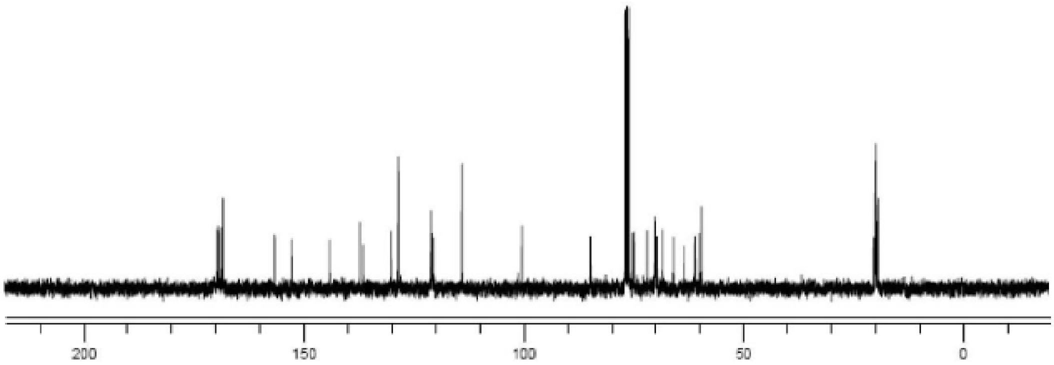


$^{13}C$ NMR spectrum of monomer **3**

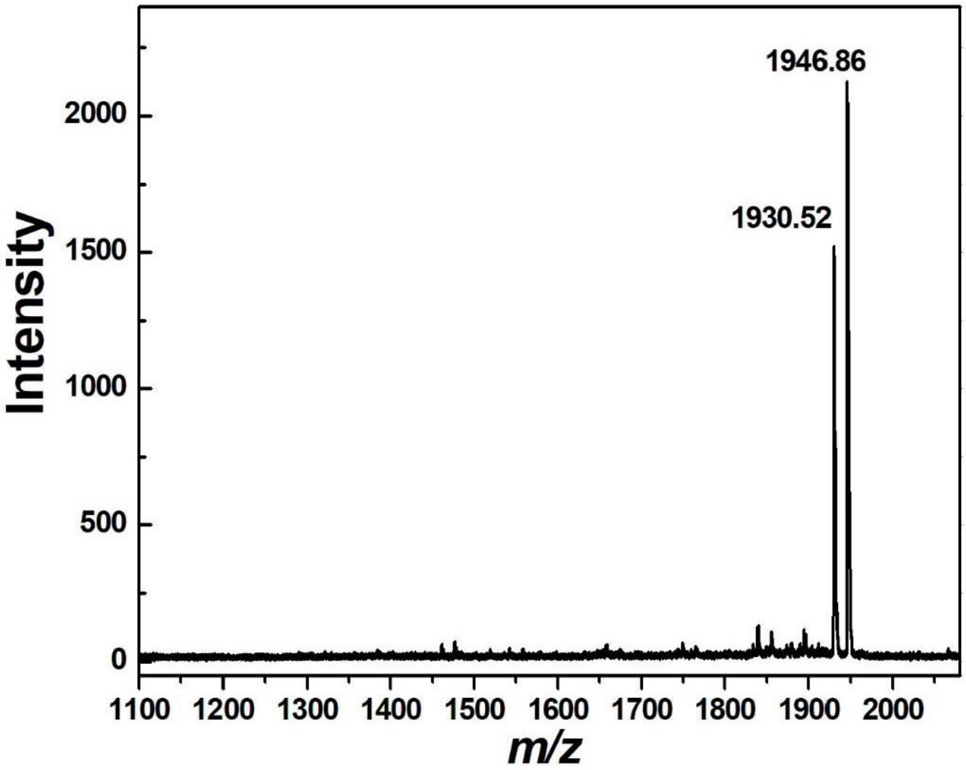

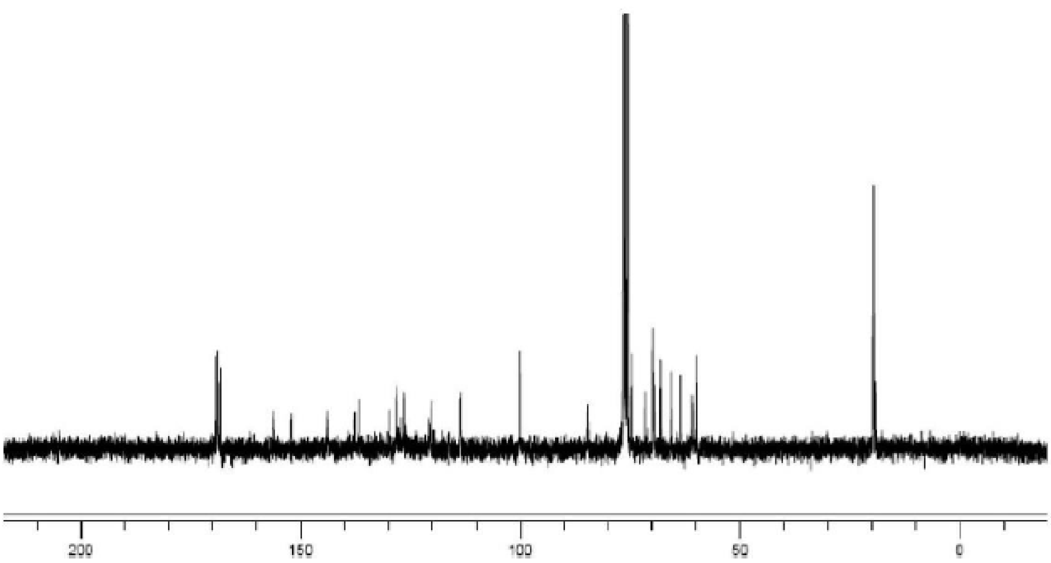


$^{13}C$ NMR spectrum of polymer **PF-AcLac**

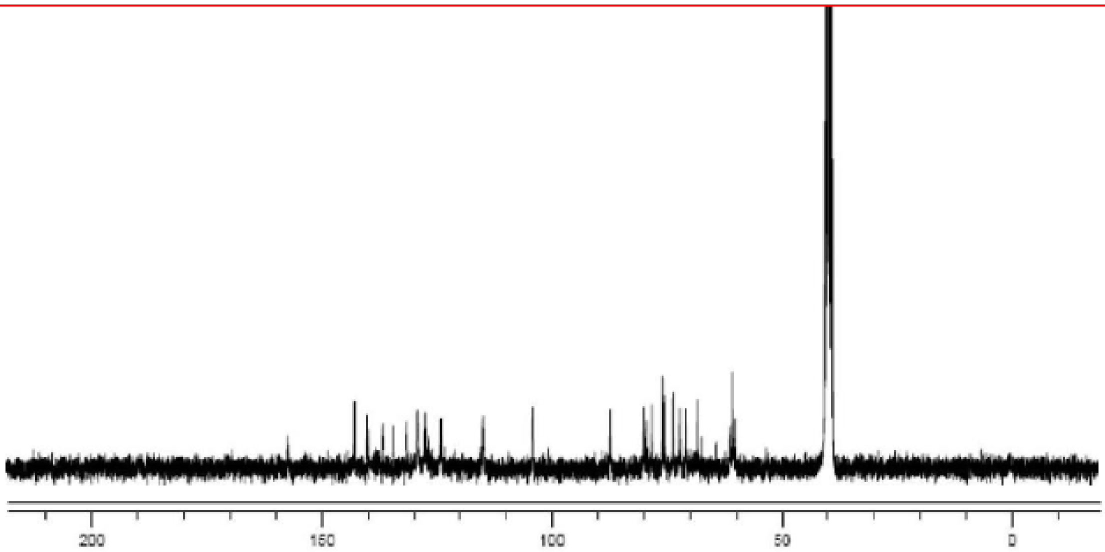


$^{13}C$ NMR spectrum of polymer **PF-Lac**
